# Determining soil water characteristic curve of lime treated loess using multiscale structure fractal characteristic

**DOI:** 10.1038/s41598-020-78489-7

**Published:** 2020-12-09

**Authors:** Xiaojun Li, Chenzhi Hu, Fengyan Li, Hongling Gao

**Affiliations:** 1 Xi’n University of Science and Technology, Xi’an, 710054 China; 2 Qinghai Bureau of Coal Geology, Xining, 810012 China

**Keywords:** Computational methods, Applied mathematics, Materials science

## Abstract

Soil–Water characteristic Curve (SWCC) is meant to describe the mechanical behavior of unsaturated soil. The present paper focuses on the internal multi-scale microstructure of Xining untreated loess and lime-treated loess with the use of scanning electron microscopy (SEM) and image processing technique. A new SWCC model was presented based on the fractal dimension of pore size distribution. The SWCC of untreated loess was calculated from fractal dimension and fitted well with curve tested from Fredlund SWCC device. The SWCC of lime-treated loess was then calculated. Two curves of Xining untreated loess and lime-treated loess have been compared and reasons for the difference have also been discussed. The results indicate that the content of large pores in lime-treated loess decreased and the content of micro-pore increased. The bracket pores were changed into cement pores. The pore fractal dimension D of Xining untreated loess is 1.39 and the pore fractal dimension D of Xining lime-treated loess is 1.53. Air-entry value of untreated loess is 12.16 kPa, while lime-treated Loess—35.15 kPa. In transition region, matric suction of lime-treated loess was in the range of 35.15 kPa ~ 4000 kPa, while matric suction of untreated loess—12.16 kPa ~ 2600 kPa. The range of the transition region in lime-treated loess is larger than that in the loess, while in the range of saturation region, the reverse applies. Under the condition of the same matrix suction, the saturation of lime-treated loess is greater than that of untreated loess. In the residual region, the difference of SWCC of soil samples is small.

## Introduction

SWCC is used to define the correlation between water content or degree of saturation with suction^1^. It is the most important and basic tool for studying soil moisture movement, adjusting and using soil water, and performing soil improvement, and it plays a very important role in the field of civil engineering^2–4^. Previous studies have highlighted the benefits of SWCC: It can determine the parameters of unsaturated soil parameters, such as shear strength, coefficient of permeability, indirectly reflect the distribution of soil pores, and judge the soil texture and the distribution of soil moisture in the suction section^5–7^. The SWCC has been widely used to analyze the stability of slopes, transient seepage, the coupling of the mechanical and hydrological effects of vegetation^8–11^.

The determination method of SWCC is divided into direct method obtained curve from the experimental method and indirect method determined curve through empirical formula. Experimental method is time-consuming, laborious, and costly, and it also has greater limitations in the measurement range. Therefore, more and more efforts have been made on indirect parameter estimation of SWCC due to those difficulties in experiments. Chin et al.^12^ proposed a method of determining the SWCC of coarse- and fine-grained soils by using one-point SWCC measurement and basic index properties. The results revealed that better SWCC estimation tends to favor at suctions of 10 and 500 kPa for two soils. Zou and Leong^13^ proposed a simple method to estimate unimodal SWCC by using an ensemble of point pedo-transfer functions. The estimated result was consistent with the experimental SWCCs, so ensemble PTF is an effective approach to estimating unimodal SWCC. Xue et al.^14^ presented a method of predicting the SWCC in the capillary and adsorption zones by using measurement data in the low suction range. Tao et al.^4^ proposed a simplified unified fractal model of relative permeability coefficient based on the classical model for evaluating relative permeability coefficient, and compared the prediction results of this model with experimental data of different types of unsaturated soils.

Due to pore size distribution that has a great influence on SWCC, a large number of attempts have been made to predict SWCC based on the pore size distribution^15,16^. Tao et al.^17^ taken Hunan clay as the research object, through the fractal dimension calculated by multifractal based on PSD, to predict the unsaturated hydraulic conductivity. Tao et al.^18^ discussed the difference in fitting effect between different fractal models and empirical models through summarized and divided the existing SWCC fractal model. In these studies, fractal theory is used to quantitatively characterize the complex pore distribution. Although fractal SWCC models have been established, the determination of the parameters used in those models is not an easy task. Previous studies have shown that both MIP and CT methods for obtaining PSD have certain flaws. MIP cannot obtain closed pores, while CT scan resolution cannot distinguish small pores. In this respect, Sun et al.^19^ fortunately proposed a SEM image processing technology to enable SEM to make up for the shortcomings of MIP and CT, and achieve multi-scale structure image acquisition. These characteristics provide a great possibility to establish a good prediction of the SWCC of loess with SEM. In addition, the previous studies were mostly focusing on the SWCC of loess, lime-treated loess has hardly been measured by using the fractal theory.

Hence, the main objective of this study is constructing a new SWCC prediction method to compare the difference of SWCC between lime-treated loess and untreated loess. This method is based on the fractal dimension obtained by using SEM technology. To achieve these aims, the following steps are designated:

1. Conducting samples preparation and electron microscope scanning.
2. Obtaining fractal characteristics of samples by using SEM images after processing.
3. Constructing the SWCC prediction method based on the fractal theory.
4. Applying the SWCC model to predict SWCC of lime-treated loess.
5. Comparing SWCC characteristics of different samples.

## Materials and methods

### Study site

**Figure 1:**
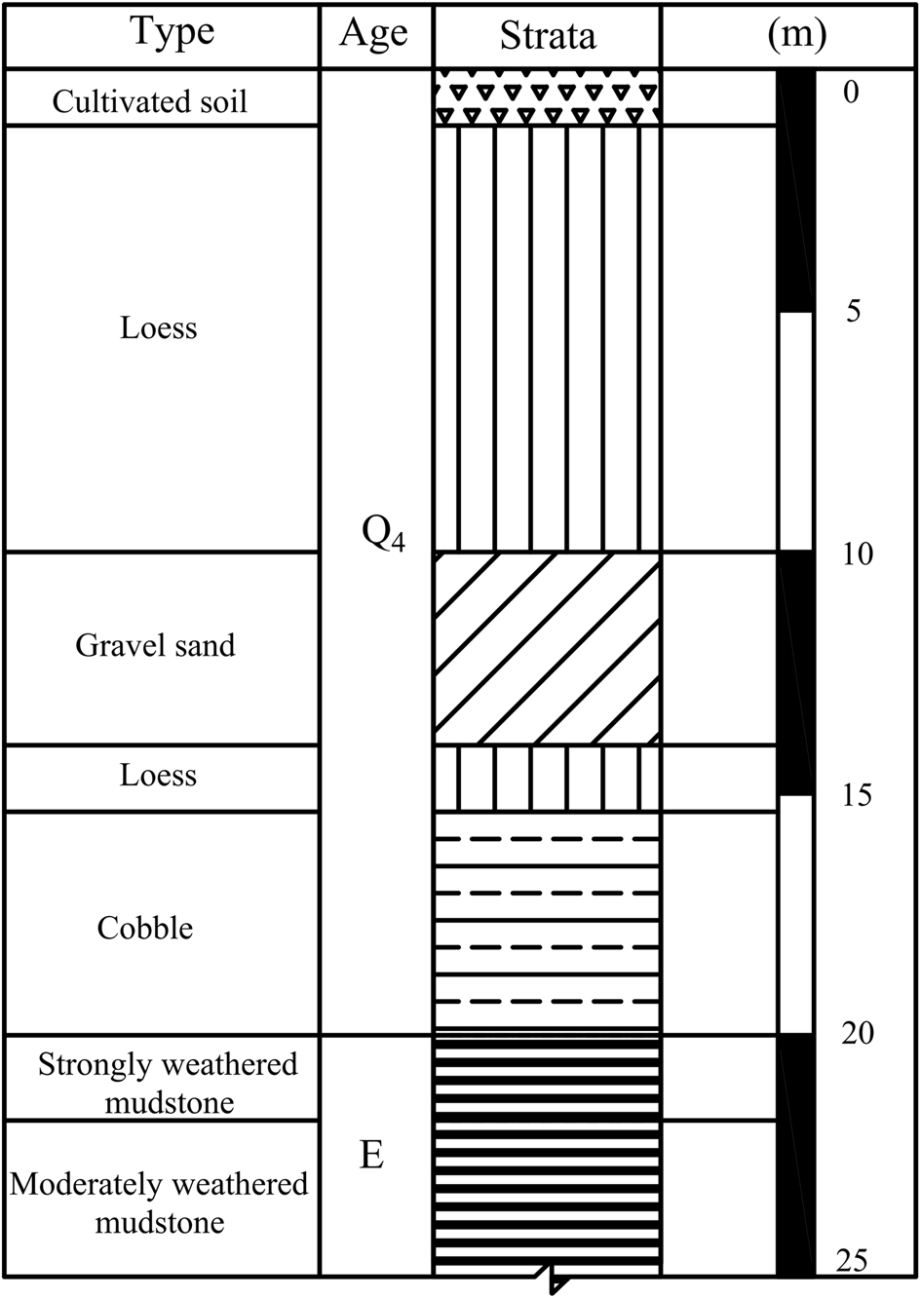
Stratigraphic profile of the study area.

The study site (101°77′ E, 36°62′ N) is located in Xining city (Qinghai Province, northwest China) and located at 2260 m above mean sea level, with relatively higher terrain in the northwest and lower terrain in the southeast. The elevation of the exploration point on the site is 2277–2279 m, and the absolute height difference is 2 m. The terrain of the site is relatively flat, with relatively higher terrain in the northwest and lower terrain in the southeast. The geomorphic unit is single, and it belongs to a terrace on the south bank of the Huangshui River. The stratigraphic profile of study site was shown in Fig. 1.

### Sampling

Loess was sampled in Qinghai-Tibet Science and Technology Museum in Xining. The percentage of silt is the highest in the samples, followed by clay. The soil is uniform and has no luster reaction. The samples of natural loess were obtained according to the following procedure. Firstly, the site of the soil sampling was determined. Then, the specimen size of 150 × 150 × 150 mm and three cylindrical samples with 63 mm in diameter and 25 mm in height were taken vertically at a depth of 2 m. To avoid any perturbation, loess samples covered plastic wraps were marked and carefully transported to the laboratory. Square sample was used to determine the main properties of the soil and the cylindrical sample was used to obtain samples of SEM and SWCC. The dried soil strength is low, with large holes in it. The main properties of the soil were shown in Table 1.

**Table 1:** Main physical properties of loess from Xining.

Parameter	w (%)	r_d_ (kN/m^3^)	G_s_	e	w_L_ (%)	w_P_ (%)	I_L_ (%)	I_P_ (%)
values	16.7	14.0	2.70	0.93	24.8	15.4	0.13	9.4

*w* Water content, *rd* Dry unit weight, *Gs* Specific gravity, *e* void, *wL* Liquidity limit, *wP* Plastic limit, *IL* Liquid index, *IP* Plasticity index.

### Measurement of micro-porosity structure based on SEM

#### Sample preparation

SEM sample included lime-treated loess and untreated loess. Samples of lime-treated loess were obtained according to the following procedure:

1. The soil was crushed, air-dried, and sieved through the aperture of 2 mm.
2. Loess and lime were mixed evenly according to a mass ratio of 100:7 to obtain lime-treated loess with the water content of 16.7% and the dry density of 1.39 g/cm^3^.

**Figure 2:**
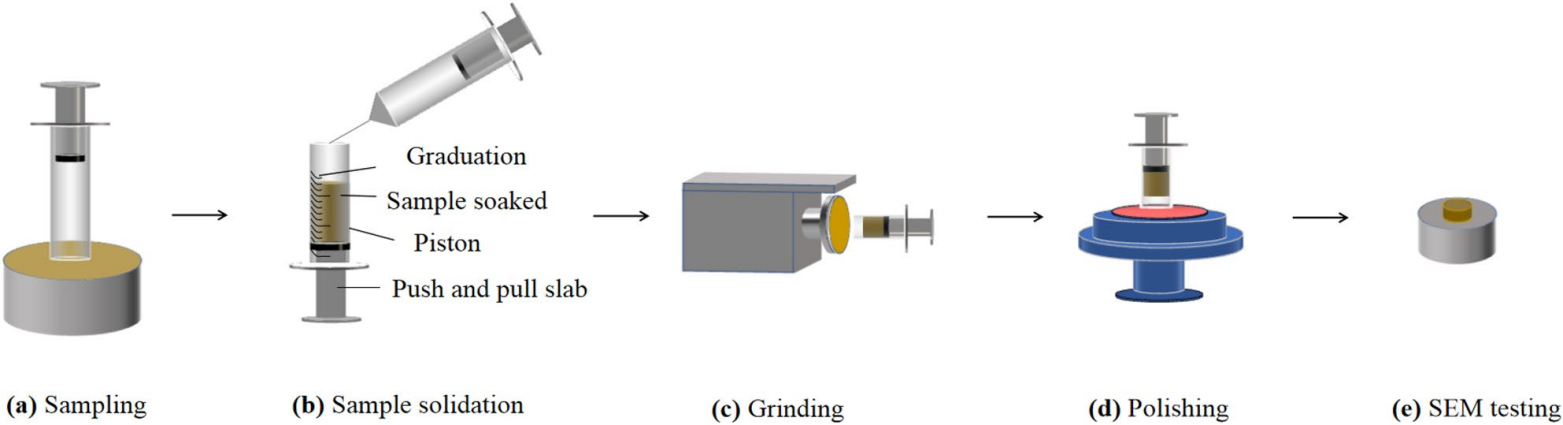
Work flow to generate the sample.

Figure 2 shows the preparation process of the SEM sample and test procedures.

1. As shown in Fig. 2a, the untreated loess sample, 10–15 mm in height, was cut from original ring knife sample to the soil extractor^20^. The lime-treated loess was pressed into the extractors and dried.
2. Prior to mixing with acetone, the epoxy resin was treated with a water bath to dilute it. Then, the epoxy resin was dissolved in acetone and stirred constantly until a clear solution was obtained. Ethylenediamine and dibutyl phthalate were added in order. In order to get the epoxy resin solution, Epoxy resin, acetone, ethylenediamine, and dibutyl phthalate were mixed thoroughly at a 50:400:3.5:1 volume ratio.
3. As shown in Fig. 2b, a needle tube was used to gently drop the epoxy resin solution into the soil extractor. The frequency is once an hour until the solution reaches saturation. After being air-dried for 24 h under natural conditions, it was placed in a constant temperature box at 40 °C for one day. Then, the temperature was adjusted to 60 °C until the sample was completely cured.
4. The sample was preliminarily polished to ensure that the two sections of the cylindrical sample are flush (Fig. 2c). Then, the sample was placed on the ground glass and polished with emery solution to smooth (Fig. 2d).
5. Next, the smooth surface of the samples was continuously polished on the polishing cloth with the polishing liquid that is configured with a 4:1 volume ratio of water to alumina powder. The time of polishing was control about 2 h. Finally, the polishing powder on the surface of the sample was cleaned with water and dried.
6. A gold sprayer was used to coat the sample (Fig. 2e). The current and time of the instrument were set to 20 mA and 110 s, respectively.

#### Sample scanning

**Figure 3:**
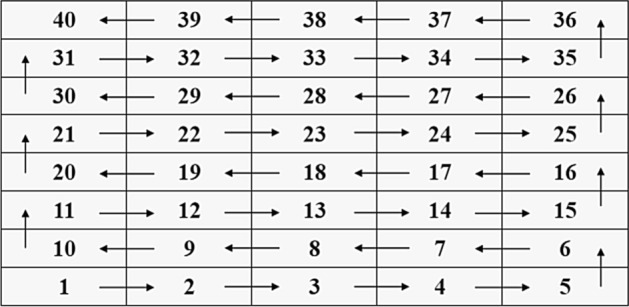
Scanning route.

**Figure 4:**
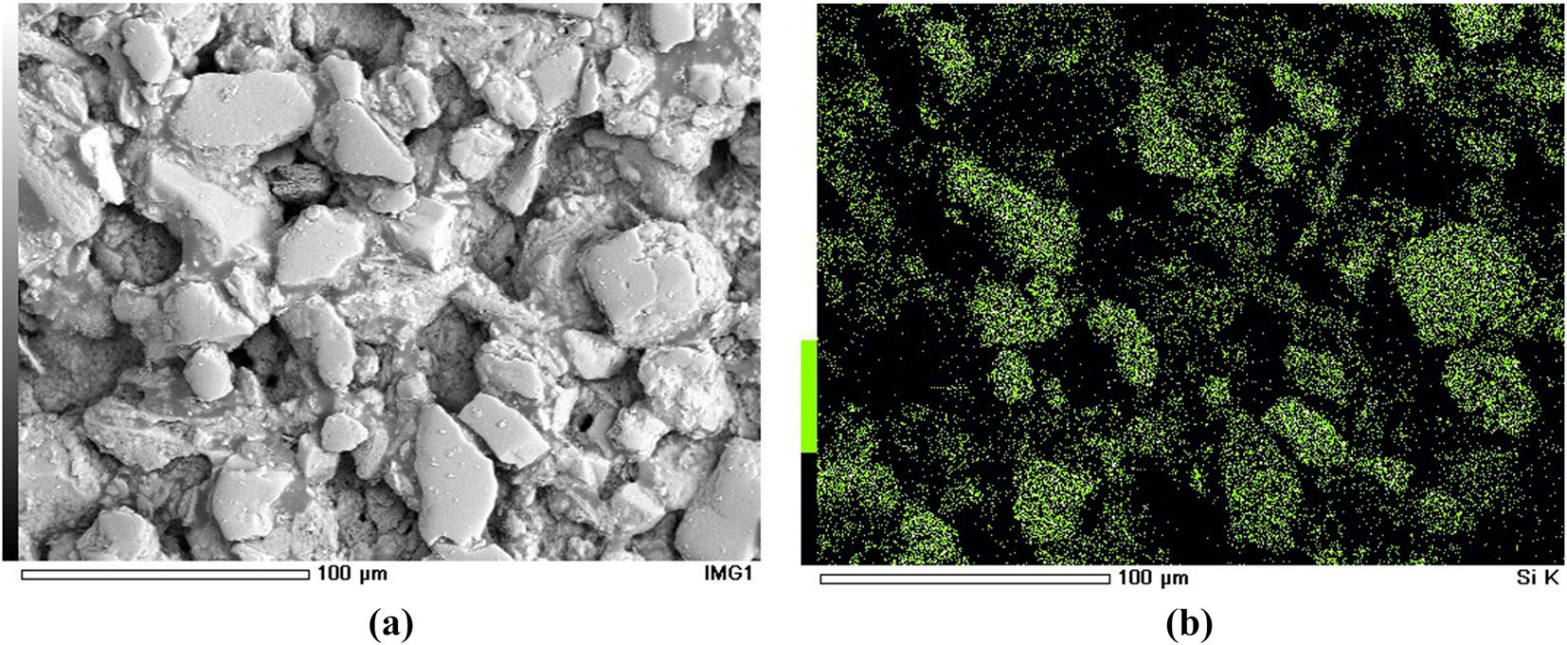
Energy spectrum map and surface scan image: (**a**) Energy spectrum map; (**b**) Surface scan image.

The samples were scanned with JSM-6390A scanning electron microscope made by JEOL Ltd0, Japan. The samples were scanned at a magnification of 500 using SEM. In order to observe the microscopic characteristics of the samples’ surface within a limited field of view, the samples were scanned in the form of the S-shaped route (Fig. 3). The No. 1 ~ No. 5 images constituted the base layer of the whole picture in such a way that adjacent images had an overlapping width of 30 μm. The samples were scanned according to a magnification of 500 times and 40 images were obtained with each sample (Fig. 4a). The surfaces of the samples were scanned to obtain the energy spectrum base map and surface scanning image of seven elements including Si, Al, Ca, K, Fe, Mg, and Na and 320 images were obtained for each sample. Figure 4b shows the surface scanned image of Si element.

### Image processing

**Figure 5:**
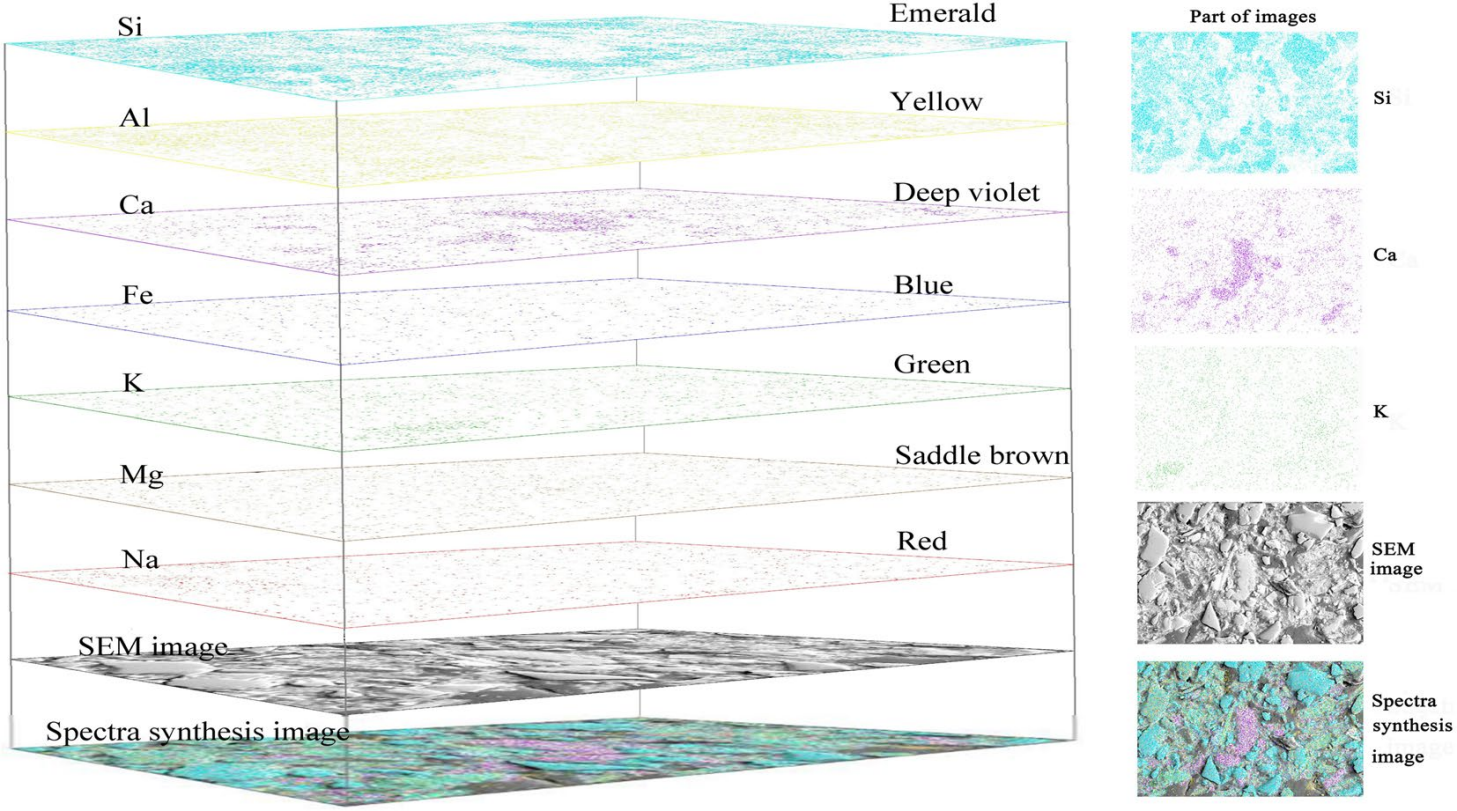
Color-added map: The corresponding colors of seven different elements were added seven different colors on the base map.

Figure 5 vividly shows the specific procedure of image processing. Image processing involves base map splicing and color overlapping. In order to ensure complete and seamless splicing, the images were spliced in the form of an S-shaped route with a horizontal overlapping width of 1/4 and a vertical overlapping length of 1/3. In the procedure of color overlapping, corresponding colors fell respectively into seven different elements, and then these seven different colors were added to the base map according to the RGB values^19,21^. Finally, color-added maps were spliced in the same way as the base maps.

### Fractal theory

Based on the image processing technology, the distribution of fractal dimension of pores was obtained by using the calculation method in Eq. (1), put forward by Xu and Fu^22^.

1 $$ D = - \mathop {\lim }\limits_{r \to 0} \frac{\ln N(r)}{{\ln r}} $$

where D is distribution of fractal dimension, r is the mean pore size, N(r) is the number of pores corresponding to r.

### Measurement of SWCC

**Figure 6:**
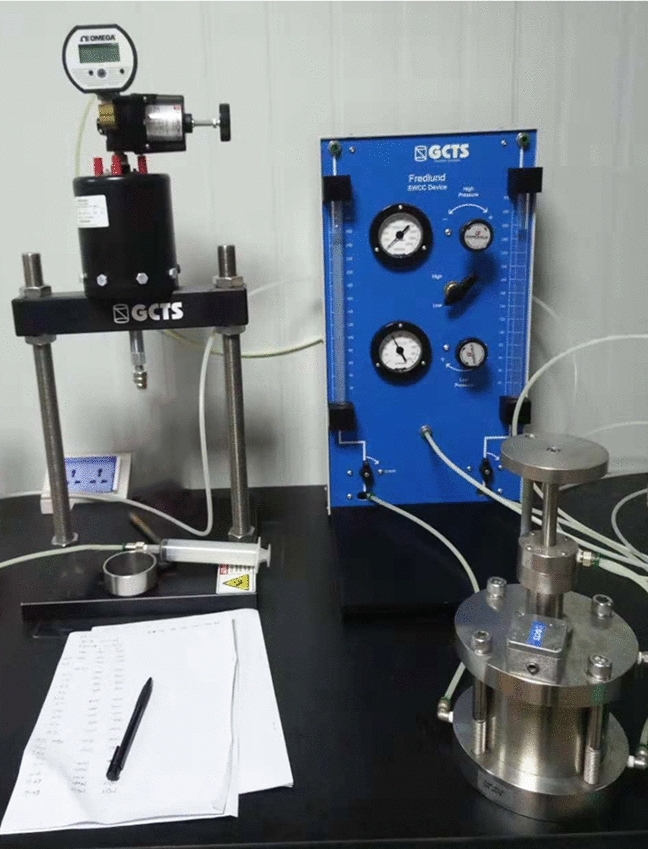
Soil–water characteristic pressure plate apparatus.

The SWCC of loess was tested by Fredlund SWCC Device (Fig. 6). Soil–water characteristic pressure plate apparatus controls pore air pressure by adjusting valve. According to the air-entry value of clay plate, pore air pressure ranges between 0 and 1500 kPa, and pore water pressure is 0 (ignoring the influence of position water head). Then the matrix suction and the change of water content in the samples could be obtained by volume change tube. The corresponding saturation under all levels of matrix suction was calculated, thus the SWCC being determined. The volume converter was marked with one scale of per 1 mm. By calibrating the reading of the volume converter, 1 mm on the left tube indicated 0.076 g water content and on the right tube − 0.069 g. The matric suction and its corresponding saturation were obtained, as is shown in Table 2.

**Table 2:** The measured matrix suction and its corresponding saturation.

Matric suction /kPa	20	40	60	80	100
Saturation/%	86.5	68.2	59.6	56.7	53.1

### The proposed method

The influence of matric suction on strength depends on the distribution of micropores. Therefore, the SWCC model was established based on the distribution characteristics of the pore.

The area of pore is calculated from the formula: $S = \int\limits_{0}^{R} {\pi r^{2} } dN$, that:

$$ \begin{gathered} S = \int\limits_{0}^{R} {\pi r^{2} } dN = \int\limits_{0}^{R} {\pi r^{2} } d(Cr^{ - D} ) \hfill \\ = - \pi CD\int\limits_{0}^{R} {r^{2} } r^{ - 1 - D} dr \hfill \\ = - \pi CD\int\limits_{0}^{R} {r^{1 - D} } dr \hfill \\ = \pi CD/(2 - D)R^{2 - D} \hfill \\ \end{gathered} $$

2 $$ S = AR^{2 - D} $$

In the formula: R is the maximum pore size in soil and $A = \pi CD/(2 - D)$.

During the process of humidification, the water is filled with small pores first, the radius is *r*, that the area filled with water is:

3 $$ S_{w} = Ar^{2 - D} $$

The saturation *S*_*r*_ is:

4 $$ S_{r} = \frac{{S_{w} }}{S} = (\frac{r}{R})^{2 - D} $$

The relationship between air entry value and pore size of unsaturated soil based on the Young–Laplace formula is:

5 $$ u_{se} = \frac{{2T_{s} \cos \alpha }}{R} $$

In the formula: *u*_*se*_ is the air entry value of unsaturated soil; *T*_*s*_ is the surface tension; *α* is the contact angle; R is the maximum radius of the soil pore.

And the relation between saturation and matric suction is:

6 $$ S_{r} = \left(\frac{{u_{s} }}{{u_{se} }}\right)^{D - 2} $$

## Results and discussion

### Analysis of size of pores

**Figure 7:**
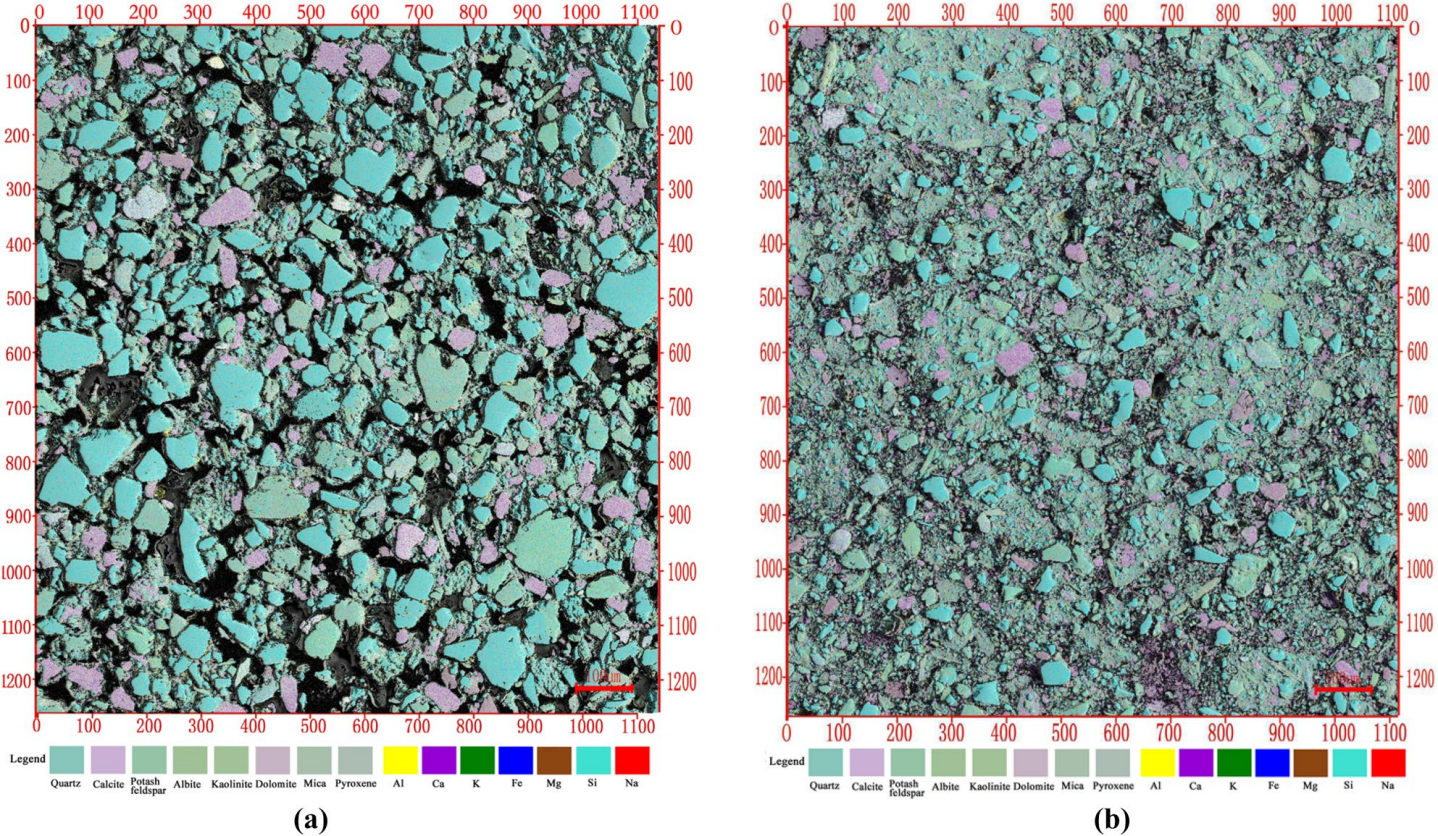
Multi-scale microstructure of untreated loess and lime-treated loess from Xining: (**a**) untreated loess; (**b**) lime-treated loess.

**Figure 8:**
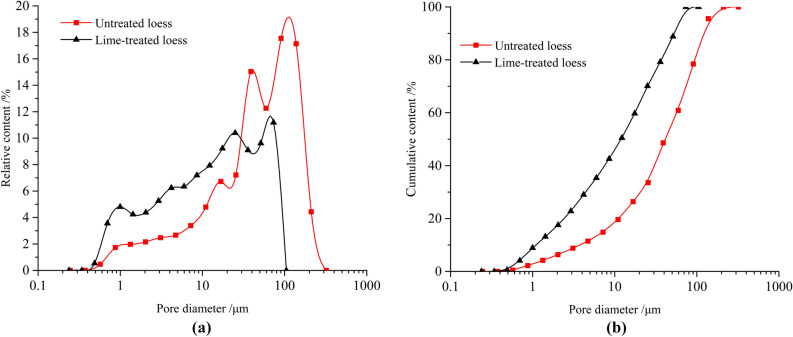
Pore distribution curve of untreated loess and lime-treated loess from Xining: (**a**) untreated loess; (**b**) lime-treated loess.

After processing the scanned image, the corresponding colors of seven different elements were added on the base map. Multi-scale microstructure mineral distributions of untreated loess and lime-treated loess were obtained (Fig. 7). Engineering geological characteristics of soils are influenced by pores structure, grain structure, and cementation. The pore size distribution is one of the main parameters quantitatively evaluated pores structure and can indicate complex pore structure characteristics in far more detail than porosity alone^23,24^ . Equal diameter of limed treated loess and untreated loess was obtained based on Multi-scale microstructure mineral distributions. Figure 8a presents the frequency curves of the pore size distribution of the two samples. Overall, the PSD curves of two samples all reveal a skewed distribution and exhibit multi-peaks. For lime-treated loess, the change of PSD curves is relatively smooth and slow while those in the untreated loess are reversed. Locally, untreated loess shows two peaks at 19% and 15%, corresponding to (113 μm) and (40 μm) of pore diameter. Lime-treated loess exhibits two peaks at 10.5% and 11.6%, corresponding to (69 μm) and (25 μm) of pore diameter. Compared with untreated loess, the lime-treated loess of the pore size distribution concentrates and shifts to a smaller radius. As is shown in Fig. 8b, the cumulative distribution curve of lime-treated loess is located above the untreated loess and is more gentle.

It can be concluded that the contents of large-and medium-sized pores in untreated loess are higher, whereas contents of micro-and-small sized pores are lower. This is consistent with the observation in the Fig. 7, and it can be intuitively observed that these pores are composed of quartz and feldspar with the size between 50–100 μm. After lime treatment, macropores and medium pores have become fewer, whereas micropores and small pores have increased gradually. The contents of micropores and macropores changed significantly. In lime-treated loess, there are no macropores and there are more cement formed by lime. It is indicated that lime mainly affects the micropore in the soil, and the amount of macropores and medium pores decrease, which transforms into smaller pores. A more stable structure around the pores came into being and the soil gradually became dense.

Table 3 provides the size and number of pores from the SEM images of untreated loess and lime-treated loess. The pore size and its amount indicate that there is a high proportion of tiny pores in lime-treated loess. According to the measured aperture range, it is divided into n = 15 aperture levels.

**Table 3:** Aperture—Quantity measurement results.

Aperture level	Loess		Lime-treated loess	
	Pore quantity N	Mean pore size (μm)	Pore quantity N	Mean pore size (μm)
1	16,626	0.57	35,586	0.49
2	13,991	0.87	33,222	0.69
3	8101	1.33	21,150	0.99
4	4442	2.03	11,597	1.43
5	2586	3.09	7194	2.04
6	1511	4.72	4567	2.92
7	927	7.19	2781	4.18
8	579	10.98	1605	5.98
9	359	16.75	952	8.56
10	205	25.55	548	12.24
11	126	38.96	310	17.51
12	57	59.43	169	25.06
13	31	90.64	83	35.85
14	12	138.24	39	51.29
15	2	210.85	16	73.39

### Distribution of fractal dimension of pores

**Figure 9:**
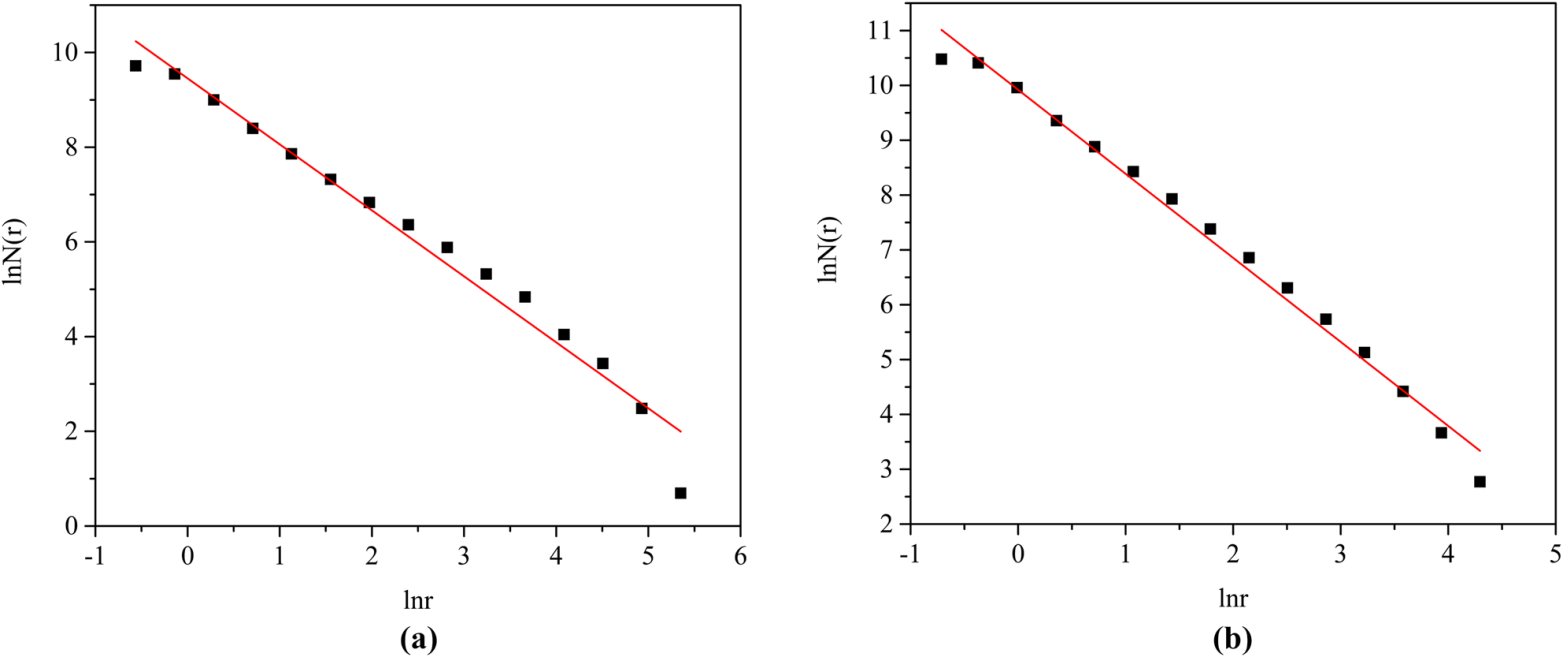
Determination of fractal dimension D: (**a**) untreated loess; (**b**) lime-treated loess.

Following the procedure presented in part of “Fractal theory”, D was directly determined from fitting the experimental data of Table 3 to Eq. 1, as shown in Fig. 9. The related fitting parameters of unsaturated soil were listed in Table 4. The correlation coefficients of all experimental data are > 0.97, which indicates that the fractal behavior of the pores is significant. The distribution of fractal dimensions of pores of untreated loess is smaller than that in lime-treated loess. It is considered that the addition of lime has reduced the average pore size, made the soil structure compact and increased the strength of the soil. Lime-treated loess has a higher fitting coefficient that the data points between 3–8 are closer to the fitting curve in Fig. 9b. The corresponding pore size range is 0.99–8.56 μm. The number of pores in the lime-treated loess is higher than that of the untreated loess in this section of pore size. It can be seen that the number of macropores affects its fractal characteristics, and macropores weaken the fractal of the pores. As the number of macropore increases, the fractal characteristics decrease. This finding suggests that the fractal characteristics of pores are controlled by the pore size distribution pore size curve, which is consistent with the results of Tao et al.^25^ using silty clay for fitting research.

**Table 4:** Distribution fractal dimension of pores in untreated loess and lime-treated loess.

Sample	Untreated loess	Lime-treated loess
Expression of Fitting Line	y = − 1.39x + 9.45	y = − 1.53x + 9.93
Correlation Coefficient R^2^	0.972	0.989
Fractal dimension	1.39	1.53

### Determine the SWCC for lime-treated loess

To predict the SWCC, the core problem in this section will be to focus on accurately predicting air-entry value. Combining Eqs. 5–6, the most important thing is to determine 2Ts cosα. Under isothermal conditions, *2T*_*s*_* cosα* can be regarded as a constant^26^. The fractal dimension of pore distribution of loess is known to be 1.39. The SWCC model of loess was determined as follows:

7 $$ S_{r} = \left(\frac{{u_{s} }}{{u_{se} }}\right)^{ - 0.61} $$

**Figure 10:**
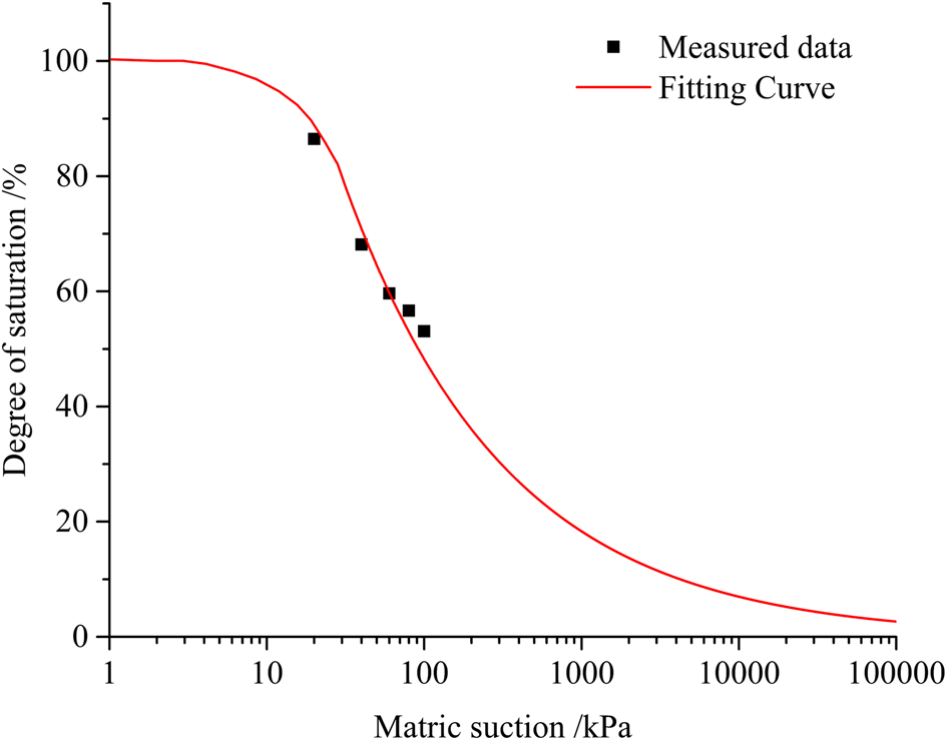
Fitting of SWCC of untreated loess.

Then, the measured matrix suction and its corresponding saturation were fitted with the SWCC model and the result is shown in Fig. 10. The measured points fall on both sides of the newly built SWCC. The air-entry value of the undisturbed loess was obtained based on the fitting curve (12.16 kPa). It is known that the maximum pore size of untreated loess is 0.211 mm. From Eq. 5, *2T*_*s*_*cosα* can be calculated as 3.714 kPa mm. The maximum pore size of lime-treated loess is 0.073 mm. The fractal dimension of pore distribution of lime-treated loess is known to be 1.53. Under isothermal conditions, the SWCC model of lime-treated loess was determined as follows:

8 $$ S_{r} = \left(\frac{{u_{s} }}{50.87}\right)^{ - 0.47} $$

Therefore, the model of SWCC established in the current study is feasible. According to Eqs. 7 and 8, the SWCC of loess and lime-treated loess can be obtained.

**Figure 11:**
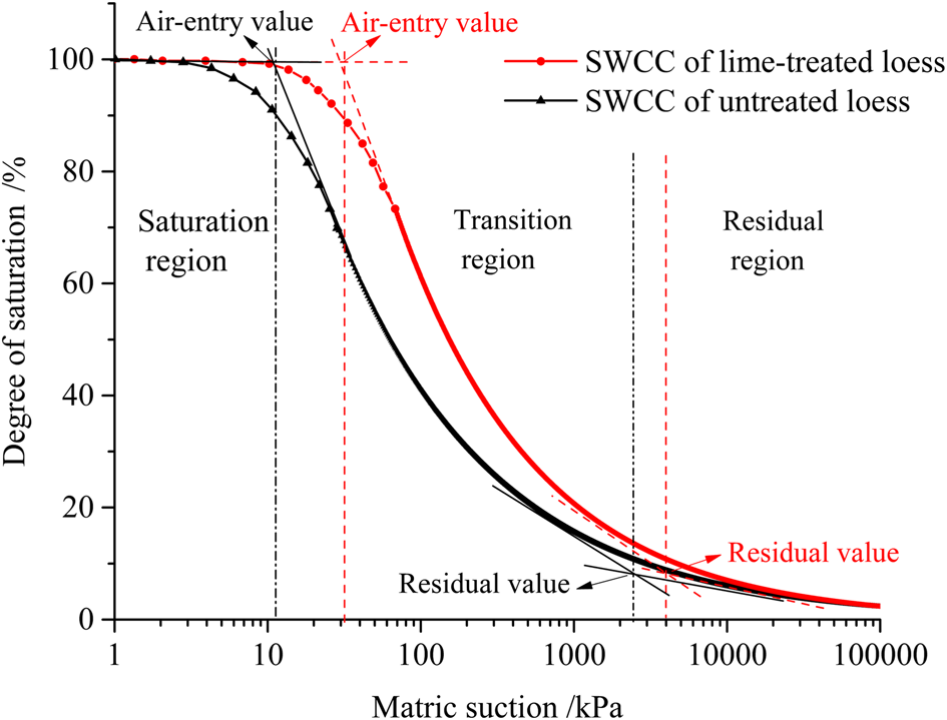
SWCC of loess and lime-treated loess.

The SWCC of lime-treated loess and untreated loess was presented in Fig. 11. The air-entry value, residual suction, residual saturation of untreated loess is 12.16 kPa, 2.46 kPa, and 0.082, respectively. The air-entry value, residual suction, residual saturation of lime-untreated loess is 50.87 kPa, 4.00 kPa, and 0.083, respectively. For air-entry value, lime-treated loess is much greater than that of untreated loess. The air-entry value of the soil refers to the pressure value required for air to start entering the soil, which is determined by the maximum pore in the soil. As is shown in Table 3, the maximum pore size of untreated loess is 0.211 mm, which is 3 times more than that of lime-treated loess. It can be seen from the Fig. 7a that there are a large number of large-grain minerals such as quartz and feldspar in the untreated loess. Those minerals overlap with each other to form a large number of macropores. The air-entry value is low due to the influence of the macropores. However, the addition of lime leads to the formation of new cementation in the treated loess to fill the pores, resulting in the destruction of the macroporous structure of the untreated loess. These results imply that addition of lime reduces the large and medium pores, and the micro and small pores increase.

For residual saturation, the lime-treated loess also shows a larger value. This is due to the water that is difficult to remove in the small pores of the soil. The soil body exhibits a stable water content, which is determined by the smallest pores of the soil and reflects the proportion of small voids to a certain extent. Yao^27^ artificially believes that the matrix suction can reach 11, 200kpa when the pore size is 0.01 microns, so the results of residual saturation in this paper are reliable.

According to the air-entry value and residual value of soil, the SWCC can be divided into saturated region, transition region, and residual region^28^. Although the soil–water characteristic curve shown develops in the same trend as a whole, the degree of change is not the same. The matrix suction range of the lime-reinforced loess transition zone is about 35.15 kPa ~ 4000 kPa, while the undisturbed loess matrix suction range is about 12.16 kPa ~ 2600 kPa. The saturated area of lime-treated loess is wider than that of untreated loess (Fig. 11). Under the same matrix suction condition, the saturation of lime-treated loess is larger than that of untreated loess. The transition region of lime-treated loess is obviously larger than that of untreated loess. In the residual region, the difference of soil–water characteristic curves between the two soils is smaller and smaller, which is similar to coincidence. The difference is mainly controlled by the pore distribution characteristics of soil samples. The smaller the average equivalent radius of pore is, the greater the matrix suction of soil sample is. With the increase of micro and small pore, the structure of soil is denser and the suction of matric is larger.

The fractal model of the SWCC is gradually established based on the fractal basic characteristics that the pore size, pore size distribution, and particle surface area of the soil have self-similarity. The model determines the fractal dimension of the soil structure and predicts the corresponding hydraulic characteristic parameter value according to the corresponding water characteristic curve fractal description model. Compare the others method, this method simplifies the difficulty of parameter determination in the empirical model to a certain extent.

## Conclusions

In this work, a multi-scale microstructure analyzing approach was used to study the effects of lime on the characteristics of loess, and a new SWCC model was presented based on the fractal dimension of pore size distribution. The following main conclusions may be drawn from the results of the present study:

1. The two types of soil have complex pore structures and obvious different characterized by number and location of peak. The content of macropores in lime-treated loess decreased and the content of micro-pore increased while those in the untreated loess are reversed.
2. The lime-treated loess has a higher fitting coefficient than untreated loess. The fractal characteristics of pores are controlled by the pore size distribution. The number of macropores affects its fractal characteristics. As the number of macropore increases, the fractal characteristics decrease.
3. The proposed method was successfully established and the estimated SWCC show a good agreement with the experimental SWCC.
4. The range of the transition region of lime-treated loess is larger than that of loess, while in the range of saturation region, the reverse applies. Under the condition of the same matrix suction, the saturation of lime-treated loess is greater than that of untreated loess. In the residual area, the difference of SWCC of different soil samples is small. The difference is mainly controlled by the pore distribution characteristics of soil samples.
